# OFFERING ACCREDITATION TO ASSISTED LIVING COMMUNITIES IN NORTH CAROLINA

**DOI:** 10.1093/geroni/igae098.0504

**Published:** 2024-12-31

**Authors:** Lea Efird-Green, Sheryl Zimmerman, Philip Sloane, David Reed, Yuke Tian, Scott Davis, John Preisser

**Affiliations:** University of North Carolina at Chapel Hill, Chapel Hill, North Carolina, United States; University of North Carolina at Chapel Hill (UNC), Chapel Hill, North Carolina, United States; University of North Carolina, Chapel Hill, North Carolina, United States; University of North Carolina, Chapel Hill, North Carolina, United States; University of North Carolina, Chapel Hill, North Carolina, United States; University of North Carolina Eshelman School of Pharmacy, Asheville, North Carolina, United States; University of North Carolina, Chapel Hill, North Carolina, United States

## Abstract

In 2021, the North Carolina Legislature approved and funded the Adult Care Home [Assisted Living] Accreditation Pilot Program. Across the state, the program’s goal is to evaluate the effectiveness of accreditation through quality outcome measures to determine whether accreditation achieves compliance with licensure requirements and improves or maintains quality of care compared to a control group. Assisted living communities were selected using stratified random sampling that accounted for geographic region and state-assigned quality rating. A total of 146 communities originally agreed to participate. Of the 73 communities randomly assigned to the accreditation arm, to date 39 (53%) either withdrew or chose to not go through the process of accreditation; 4 (5%) withdrew after beginning the accreditation process; 24 (33%) achieved accredited status; and 6 (8%) are delayed in completing the process or not likely to do so. The key reason for withdrawal from the accreditation process is the time and effort required in the context of staffing shortages, which has implications for long-range implementation even if accreditation is found to be beneficial. This session will present the aims and methods of the evaluation; the accreditation process itself (including benefits, potential administrative burden, and types of deficiencies found during surveys); the feasibility of becoming accredited for diverse participating communities; and potential strategies to bolster capacity for accreditation. (Additional contributors to this and the outcome presentation include Teresa Hoosier [ACHC], Jeff Horton [NCSLA], Frances Messer [NCALA], Barbara Sylvester [ACHC], and London Grantham, Aja Johnson, Danielle Owens, Toni Parker, and Katrice Perry [UNC].)

